# CholedochoClip: A Case of Obstructive Jaundice 14 Years after Cholecystectomy

**DOI:** 10.1155/2019/8038469

**Published:** 2019-03-19

**Authors:** Grace E. Kim, John D. Morris, Peter E. Darwin

**Affiliations:** ^1^Department of Internal Medicine, University of Maryland Medical Center, Baltimore, MD 21201, USA; ^2^Division of Gastroenterology and Hepatology, University of Maryland Medical Center, Baltimore, MD 21201, USA

## Abstract

Gallstone disease is a common gastrointestinal pathology that may result in surgical intervention. While cholecystectomy has relatively minimal risks, surgical clip migration is a rare complication that can cause severe morbidity and mortality. This report describes a rare phenomenon of a biliary stone forming onto a migrated surgical clip 14 years after cholecystectomy causing an obstructive jaundice. This case illustrates the importance of keeping a migrated surgical clip on the differential when encountering patients with symptoms of cholangitis after cholecystectomy.

## 1. Introduction

Gallstone disease is one of the most common gastrointestinal pathologies in the USA, and cholecystectomy remains the standard procedure for symptomatic stones. In this procedure, several surgical clips are placed to occlude cystic duct after gallbladder is removed to prevent biliary leaks. Laparoscopic cholecystectomy is the gold standard, embodying many advantages compared to open cholecystectomy including lower rates of complications, decreased length of hospitalization, and overall cost-effectiveness [1]. There still are risks including duct injury, bleeding, ventral hernia, bile leakage, and surgical site infection [2]. This case report delineates one of the rarer complications from a new stone formation on a surgical clip placed 14 years prior that migrated distally into the biliary duct causing obstructive jaundice.

## 2. Case Report

A 74-year-old female with history of hypertension and hyperlipidemia presented with a postprandial, nonradiating epigastric abdominal pain for several days. The pain came intermittently and lasted for hours at a time with spontaneous resolution. She denied fevers or chills but did endorse nausea and emesis. Her surgical history included a remote history of appendectomy decades ago and cholecystectomy 14 years ago. Patient is a never-smoker and denied drinking alcohol.

On presentation, she had scleral icterus with maximum total bilirubin of 3.9 mg/dL (reference range 0.1-1.4 mg/dL), alkaline phosphatase of 218 IU/L (reference range 30-140 IU/L), and aspartate aminotransferase (AST) and alanine aminotransferase (ALT) of 410 IU/L and 225 IU/L (reference range 7-40 IU/L and 10-65 IU/L). She had normal lipase of 23 U/L (reference range 10-51 U/L) and amylase of 60 U/L (reference range 25-115 U/L). White blood cells were also within normal limits at 6.4 x10^9^/L (reference range 4.0 – 10.8 x10^9^/L)

**Figure 1:**
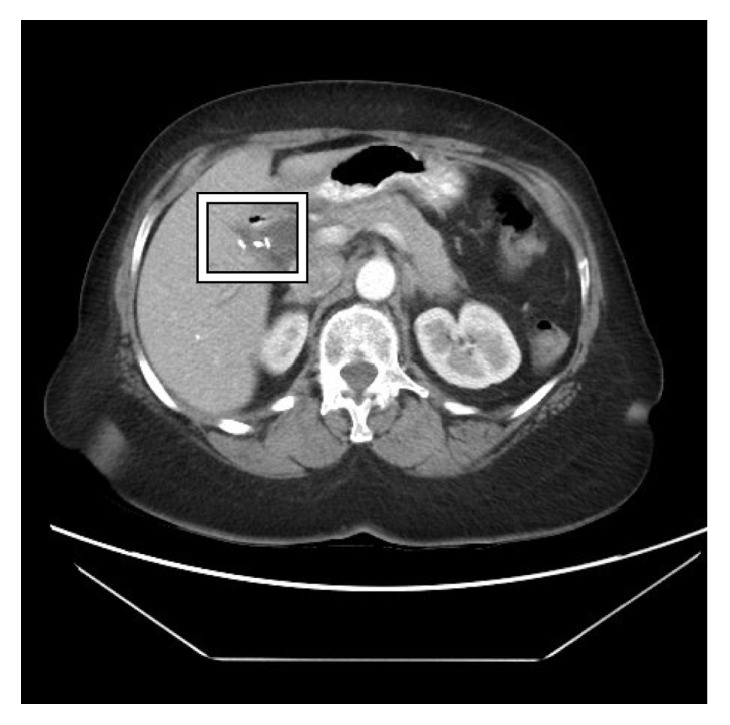
Abdominal CT showing the surgical clips by the cystic duct from the patient's prior cholecystectomy in 2004 (boxed).

**Figure 2:**
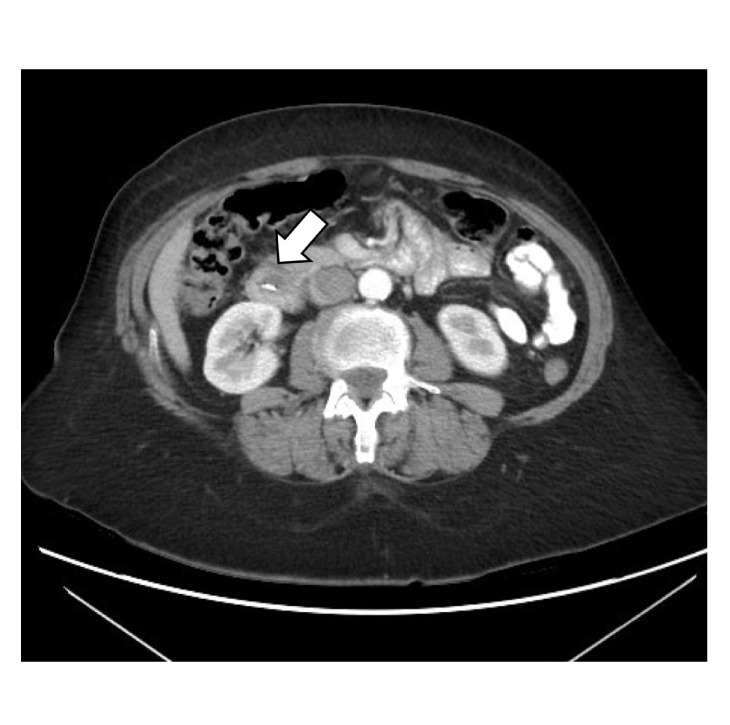
Abdominal CT showing a radiodense foreign body by the ampulla of Vater, most likely a migrated surgical clip from the prior cholecystectomy (arrow).

**Figure 3:**
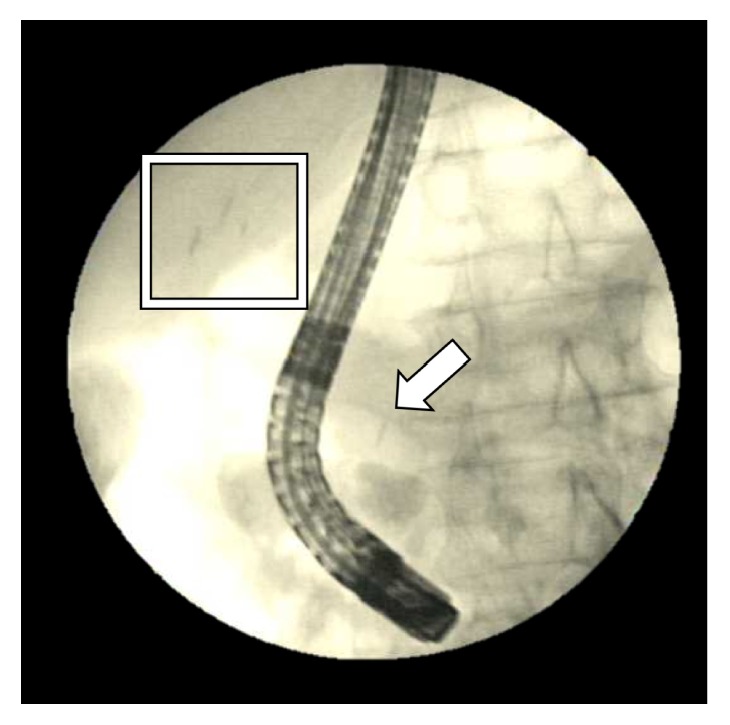
ERCP showing both the surgical clips by the cystic duct (boxed) as well as a foreign body that looks like a clip by the ampulla of Vater (arrow).

**Figure 4:**
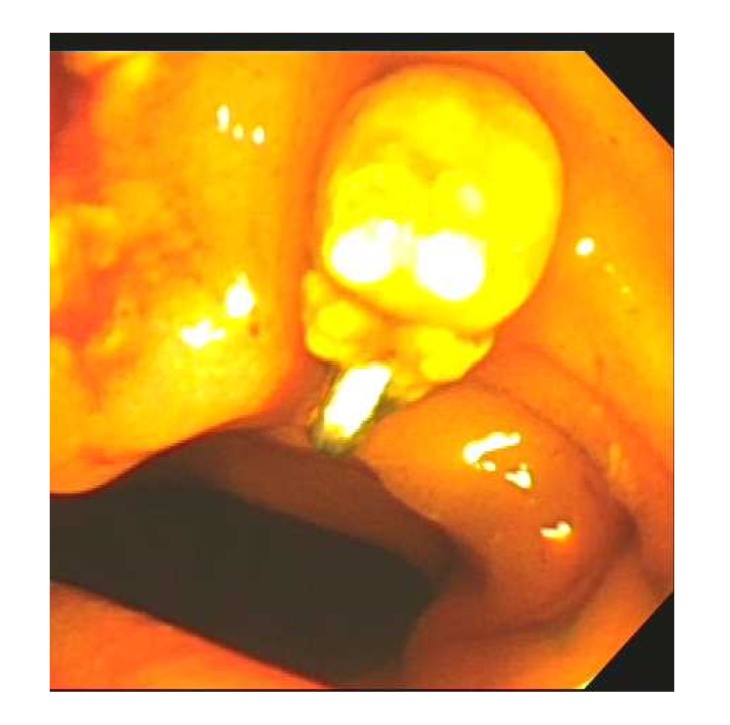
Image taken from ERCP showing a clip with stone attached, coming out of the ampulla of Vater.

She was initially started on ampicillin-sulbactam. Computed tomography (CT) of abdomen and pelvis with contrast read “marked intra- and extrahepatic biliary dilatation down to the level of the ampulla. Radiopaque foreign bodies are seen within the duodenum adjacent to the ampulla which could be obstructing.” Liver and pancreas were both normal appearing with no masses. Gastroenterology service was consulted who recommended magnetic resonance cholangiopancreatography (MRCP) given lab findings suggestive of biliary obstruction, concerning for a stone or stricture. MRCP read that “there are 2 linear metallic (and less likely calcific) densities within the duodenum at the ampulla of Vater. These appear to represent metallic surgical clips rather than stones. Unfortunately, there is magnetic susceptibility artifact off of these obscuring adjacent structures, including the distal common bile duct. There is dilatation of the more proximal common bile duct measuring approximately 12 mm. There is mild-moderate intrahepatic ductal dilatation.” Figures 1 and 2 show images from the CT surgical clips from cholecystectomy by the cystic duct (Figure 1) as well as common bile duct dilation with a hyperdense foreign body by the ampulla of Vater, most likely a migrated surgical clip (Figure 2). Endoscopic retrograde cholangiopancreatography (ERCP) was then performed, which located the surgical clip that migrated distally (Figure 3). The clip with a stone attached was then removed via balloon sweep (Figure 4), resulting in subsequent resolution of abdominal pain and normalization of the liver function tests.

## 3. Discussion

Cholecystectomy is a relatively benign procedure that is widely used. However, it does carry some risks of complications including biliary leakage and surgical clip migration. Gallstones forming on foreign bodies such as silk sutures have been reported as early as 1897 [3], with the first case of biliary stone formation from a migrated surgical clip reported in 1979 [4]. Surgical clip migration after laparoscopic cholecystectomy has been reported since 1992 [5], however the absolute number of this complication is low, accounting for less than 100 cases worldwide despite the increasing number of laparoscopic cholecystectomy procedures [6, 7]. While some clip migrations occur spontaneously without any symptoms, many of them result in biliary stricture, cholangitis, acute pancreatitis, choledochoduodenal fistula, and other severe, potentially fatal complications if not discovered in a timely manner [6, 8]. The most optimal approach to a migrated surgical clip causing obstruction is early detection and ERCP with sphincterotomy and clip removal, which provides both diagnostic and therapeutic measures to relieve the obstruction [6, 9].

Moreover, another rarer phenomenon depicted in this case is that the stone had formed onto the clip which potentiated the clinical presentation of obstructive jaundice. The stone seen on ERCP (Figure 4) had the clip almost embedded in the stone, suggesting that the stone had formed de novo around the edge of the clip over the years after the clip was placed. While the exact mechanism of stone formation after cholecystectomy is not well-understood, it is a complex, multifactorial process most likely due to the resultant stagnant biliary flow from the insertion of surgical clip in the setting of local inflammation and necrosis that initially prompted cholecystectomy [7, 10]. This in turn allows the clip to serve as a nidus for biliary stone development. Other factors include the number of clips used intraoperatively as well as incorrect placements of the clip causing incomplete occlusion of the cystic duct causing biliary leakage or partial occlusion of the common bile duct. These can further induce chronic inflammation around the biliary tree, enabling the clip to erode and migrate into the cystic and common bile ducts.

Albeit postsurgical clip migration has been well-published in the surgical literature [2, 10–12], it is nonetheless a rare complication and easily neglected in both medical and surgical fields [2, 13]. One review reported that the median time for a clip migration to be clinical evident after cholecystectomy is 2 years [6]; and since majority of these migrations occur within the first year or two of cholecystectomy [7, 10], it is even rarer to find a delayed complication from it. This could explain why both the CT and MRCP reads were rather vague about the foreign body located by the ampulla. Although there are some case reports of surgical clip migration years after cholecystectomy [14, 15], this is the first case of such finding reported from USA with such a complication from cholecystectomy performed over a decade ago.

Novel ways to mitigate postsurgical clip migration complications after cholecystectomy have been proposed in the surgical literature, including a total clipless cholecystectomy via harmonic sealing or ultrasound dissection of the cystic duct and artery [16, 17]. These approaches are still under active investigation and in the meantime, laparoscopic cholecystectomy remains the preferred procedure.

This case aims to raise awareness that the history of cholecystectomy does not rule out a biliary etiology when a patient presents with obstructive jaundice or biliary colic. This case also illustrates the importance of keeping a migrated surgical clip as well as a possible stone formation on the differential when encountering patients with cholangitis symptoms after a history of cholecystectomy.
